# Travel-associated hepatitis A in Europe, 2009 to 2015

**DOI:** 10.2807/1560-7917.ES.2018.23.22.1700583

**Published:** 2018-05-31

**Authors:** Julien Beauté, Therese Westrell, Daniela Schmid, Luise Müller, Jevgenia Epstein, Mia Kontio, Elisabeth Couturier, Mirko Faber, Kassiani Mellou, Maria-Louise Borg, Ingrid Friesema, Line Vold, Ettore Severi

**Affiliations:** 1 European Centre for Disease Prevention and Control (ECDC), Stockholm, Sweden; 2 Austrian Agency for Health and Food Safety, Vienna, Austria; 3 Statens Serum Institut, Copenhagen, Denmark; 4 Health Board, Tallinn, Estonia; 5 National Institute for Health and Welfare, Helsinki, Finland; 6 Santé Publique France, Saint-Maurice, France; 7 Robert Koch Institut, Berlin, Germany; 8 Hellenic Centre for Disease Control and Prevention, Athens, Greece; 9 Infectious Disease Prevention and Control Unit, Msida, Malta; 10 National Institute for Public Health and the Environment, Bilthoven, The Netherlands; 11 Norwegian Institute of Public Health, Oslo, Norway; 12 Karolinska Institutet, Stockholm, Sweden

**Keywords:** Europe, food-borne infections, viral infections, hepatitis A, hepatitis A virus, surveillance, travel, epidemiology

## Abstract

Travel to countries with high or intermediate hepatitis A virus (HAV) endemicity is a risk factor for infection in residents of countries with low HAV endemicity. **Aim:** The objective of this study was to estimate the risk for hepatitis A among European travellers using surveillance and travel denominator data. **Methods:** We retrieved hepatitis A surveillance data from 13 European Union (EU)/ European Economic Area (EEA) countries with comprehensive surveillance systems and travel denominator data from the Statistical Office of the European Union. A travel-associated case of hepatitis A was defined as any case reported as imported. **Results:** From 2009 to 2015, the 13 countries reported 18,839 confirmed cases of hepatitis A, of which 5,233 (27.8%) were travel-associated. Of these, 39.8% were among children younger than 15 years. The overall risk associated with travel abroad decreased over the period at an annual rate of 3.7% (95% confidence interval (CI): 0.7–2.7) from 0.70 cases per million nights in 2009 to 0.51 in 2015. The highest risk was observed in travellers to Africa (2.11 cases per million nights). Cases more likely to be reported as travel-associated were male and of younger age (< 25 years). **Conclusion:** Travel is still a major risk factor for HAV infection in the EU/EEA, although the risk of infection may have slightly decreased in recent years. Children younger than 15 years accounted for a large proportion of cases and should be prioritised for vaccination.

## Background

Hepatitis A is one of the most common causes of food-borne infection worldwide [1]. The disease spreads by faecal-oral transmission either by consumption of contaminated food or person-to-person contact [2]. Although the global burden of hepatitis A has decreased in the past 20 years [3], the World Health Organisation (WHO) still defines most of the world’s low- and middle-income countries as experiencing high or intermediate hepatitis A virus (HAV) endemicity [4]. In Europe, the incidence of hepatitis A decreased substantially [5,6] and most countries in the European Union (EU)/ European Economic Area (EEA) currently experience low or very low HAV endemicity [7]. In such populations with an increasing proportion of susceptible individuals, travel is an important risk factor for infection [2,6]. European travellers to countries with high HAV endemicity are often infected when consuming contaminated food items or drinks during travel [8,9]. A similar epidemiology is observed in other high-income countries such as the United States (US) where ca 40% of all hepatitis A cases are travel-associated [10]. 

The WHO and most EU/EEA countries recommend HAV vaccination to European travellers visiting countries with intermediate or high endemicity [11]. Nevertheless, findings from a recent survey among overseas travellers from France, Germany, Italy, Spain, and the United Kingdom (UK) suggested a moderate vaccine coverage against HAV with only half of the occasional travellers visiting endemic countries vaccinated [12]. 

A population-based study carried out in Sweden showed that the highest risk was associated with travel to East Africa, the Middle East and the Indian subcontinent [13]. The highest risk was found among young children visiting friends and relatives, as in another study carried out in the Netherlands [14]. It is difficult to compare findings from studies performed at national level for several reasons, including different data sources, populations and methodologies. Pooling data from several countries improves statistical power and allows for broader generalisation of the findings.

The objectives of this study were to describe the epidemiology of travel-associated hepatitis A, to estimate the risk for hepatitis A among travellers from EU/EEA countries and to identify groups at higher risk to help target and prioritise interventions.

## Methods

### Hepatitis A data

Surveillance of hepatitis A at the EU/EEA level is carried out by the Food- and Waterborne Diseases and Zoonoses Network (FWD-Net) under the coordination of the European Centre for Disease Prevention and Control (ECDC). The network comprises all 28 EU Member States, Iceland and Norway, which annually report hepatitis A cases among their residents to the European Surveillance System (TESSy) database hosted by ECDC. Cases are reported with a set of variables including age, sex, importation status and probable country of infection. 

A travel-associated case of hepatitis A is defined as any case reported as imported, i.e. infected following exposure outside the reporting country during a time compatible with the incubation period. For the purpose of this analysis, we included data from 13 EU/EEA countries (Austria, Denmark, Estonia, Finland, France, Germany, Greece, Hungary, Luxembourg, Malta, the Netherlands, Norway and Portugal) for the years 2009 to 2015. These countries (total population: 223 million) have comprehensive and compulsory surveillance systems for hepatitis A and reported case-based hepatitis A data for at least 80% of cases with known importation status during the study period. We chose this threshold arbitrarily to obtain reliable estimates for the proportion of travel-associated cases. In Denmark, Luxembourg and Portugal, surveillance was based on physician reporting only, whereas it was based on both laboratory and physician reporting in other countries [15]. The participating countries follow WHO recommendations as most EU/EEA countries do, i.e. they vaccinate groups at increased risk of infection. In addition, Greece has since 2008 provided universal vaccination to all children older than 12 months [7]. 

We included cases with known importation status who met the EU case definition criteria for a confirmed case of hepatitis A, i.e. any person meeting the clinical criteria and the laboratory criteria (detection of HAV nucleic acid in serum or stool or HAV-specific antibody response or detection of HAV antigen in stool) [16].

### Travel data

We obtained travel denominator data for the period from 2009 to 2015 from the Statistical Office of the European Union (Eurostat) [17]. We used the total number of nights spent, by destination country, which includes all nights that EU/EEA residents, aged 15 or older, spent in a collective accommodation establishment or in private tourist accommodation for personal or professional purposes. In most countries, this information is collected through household surveys. The collection of data should conform to the specifications described in the *Methodological manual for tourism statistics* [18]. A country may apply weighting procedures according to the sampling design used. Since travel nights were not available for all combinations of the different countries of residence with the different destination countries for all years, we estimated the total number of nights from the mean number of nights for the available years multiplied by 7 (study period of 7 years).

### Analysis

We compared travel-associated cases of hepatitis A with non-travel-associated cases for main characteristics. We defined the risk for travel-associated hepatitis A as the rate of travel-associated cases per million nights spent in a given destination (region or continent) by all travellers from the reporting country. We excluded France from the analysis by destination because information on the probable country of infection was not available. We also excluded Estonia and Norway for this part of the analysis because travel data were missing for some destinations. We considered five main regions as destinations (Africa, America, Asia, Europe and Oceania). Following Eurostat grouping, we divided America further into (i) North America and (ii) Central and South America (including Mexico). In Europe, we distinguished EU countries from other European countries (including Turkey). We calculated 95% confidence intervals (CI) for risk, assuming a Poisson distribution. To assess the trend in the annual risk for travel-associated hepatitis A over time, we estimated the annual rate of change and its 95% CI using a log-linear regression of risk for travel-associated hepatitis A associated with overseas travel in the period from 2009 to 2015.

We compared continuous variables across strata by Mann–Whitney U test. We compared categorical variables using the chi-squared or Fisher’s exact tests with a significance level of less than 0.05.

To further characterise travel-associated hepatitis A, we assessed the association of age and sex with the cases’ travel status (dependent variable) in a logistic regression analysis and estimated adjusted odds ratios (OR) and 95% CI.

### Ethics statement

Hepatitis A is part of the 52 communicable diseases for which ECDC routinely collects, analyses and disseminates surveillance data as stated by the Article 3 of its founding regulation. TESSy data are pseudonymised and processed for public interest in the area of public health. Informed consent was not required.

## Results

In the period from 2009 to 2015, the 13 participating countries reported 20,556 confirmed cases of hepatitis A, of which 18,839 (91.6%) had known travel status. Of these 18,839, 5,233 (27.8%) were travel-associated (Table 1). Four countries had proportions of travel-associated cases below 25%: Hungary (1.1%), Greece (11.3%), Estonia (17.0%) and Malta (22.7%). Of the 5,233 travel-associated cases, 2,581 (49.3%) were reported with available information on the probable country of infection. When excluding France, information on probable country of infection was available for 2,581 (94.8%) of 2,723 travel-associated cases.

**Table 1:** Main characteristics of reported cases of hepatitis A by travel status, and adjusted predictors of travel-associated hepatitis A, 13 EU/EEA countries^a^, 2009−2015 (n = 18,839)

Characteristic	Non-travel-associated cases		Travel-associated cases		Univariate logistic regression	Multivariable logistic regression^b^
	n	%	n	%	OR (95% CI)	OR (95% CI)
Total	13,606	100	5,233	100	Not included	Not included
Sex						
Male	7,356	54.1	2,907	55.6	1.06 (0.99–1.13)	1.10 (1.02–1.18)
Female	6,240	45.9	2,325	44.4	1	1
Unknown^c^	10	Not included	1	Not included	Not included	Not included
Age group (years)						
< 5	712	5.2	483	9.2	2.05 (1.79–2.34)	1.59 (1.38–1.83)
5–14	3,230	23.7	1,600	30.6	1.50 (1.37–1.63)	1.38 (1.25–1.52)
15–24	2,206	16.2	958	18.3	1.31 (1.19–1.45)	1.45 (1.30–1.62)
25–44	3,714	27.3	1,230	23.5	1	1
45–64	2,481	18.2	803	15.3	0.98 (0.88–1.08)	0.80 (0.72–0.89)
≥ 65	1,262	9.3	159	3.0	0.38 (0.32–0.45)	0.24 (0.20–0.29)
Unknown^c^	1	Not included	0	Not included	Not included	Not included
Outcome						
Alive	8,791	99.9	2,433	99.8	Not included	Not included
Dead	8	0.1	4	0.2	Not included	Not included
Unknown^c^	4,807	Not included	2,796	Not included	Not included	Not included

CI: confidence interval; OR: odds ratio.

^a^ Austria, Denmark, Estonia, Finland, France, Germany, Greece, Hungary, Luxembourg, Malta, the Netherlands, Norway, Portugal.

^b^ Adjusted for year and reporting country with France as reference.

^c^ Percentages exclude the unknown.

### Demographics

Of the 5,232 travel-associated cases with known sex, 2,907 (55.6%) were male giving a male-to-female ratio of 1.3:1 (Table 1). There was a non-significant difference between the proportion of travel-associated cases in male compared with female cases (28.3% vs 27.2%; p = 0.07). The male-to-female ratio was lower in younger and older age groups (1.1:1 below 15 years and 1.0:1 at 65 years and above), peaking at 1.5:1 for those 25–44 years of age. Of the 5,233 travel-associated cases, 2,083 (39.8%) were children younger than 15 years. The proportion of travel-associated cases in that age group was below 30% in Malta (17.0%), Hungary (17.0%) and Estonia (4.4%). Of the 2,437 travel-associated case with known outcome, four died (0.2%).

### Trend and seasonality

**Figure 1:**
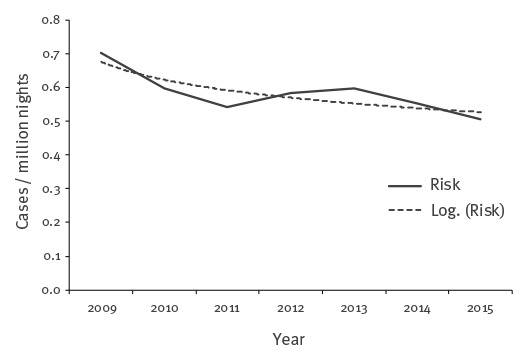
Risk of travel-associated cases of hepatitis A by million nights, 11 EU/EEA countries^a^, 2009−2015 (n = 4,705)

^a^ Austria, Denmark, Estonia, Finland, France, Germany, Greece, Hungary, Luxembourg, Malta, Portugal.

Over the study period, the average annual number of travel-associated cases was 748 (range: 637–867). The highest numbers of travel-associated cases were observed in 2009 and 2013 with 867 and 795 cases, respectively. Annual numbers of nights spent abroad were available for all countries and all years except for the Netherlands (only years 2012−15) and Norway (only years 2009−11). Between 2009 and 2015, for the 11 countries with available data on nights spent for all years, the overall annual risk for hepatitis A associated with overseas travel was 0.58 cases per million nights. The logarithmic trendline showed a decrease at an annual rate of 3.7% (95% CI: 0.7–2.7) from 0.70 cases per million nights in 2009 to 0.51 in 2015 (Figure 1). 

**Figure 2:**
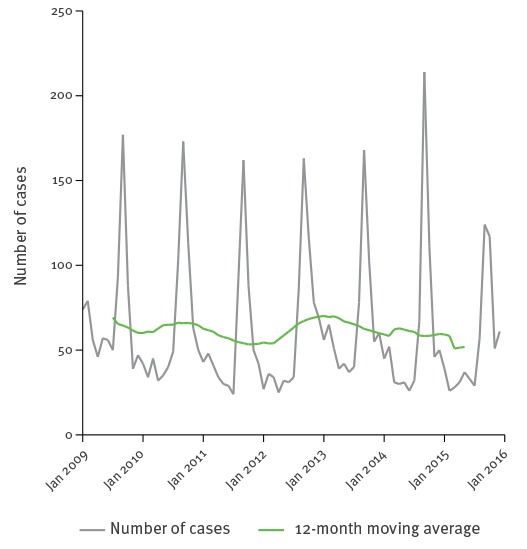
Seasonality of travel-associated cases of hepatitis A, 13 EU/EEA countries^a^, 2009−2015 (n = 5,218)

^a^ Austria, Denmark, Estonia, Finland, France, Germany, Greece, Hungary, Luxembourg, Malta, the Netherlands, Norway, Portugal.

Of the 5,218 travel-associated cases with known month of reporting, 2,016 (38.6%) were reported between August and October, including 1,181 (22.6%) in the month of September alone (Figure 2). Of the 2,078 travel-associated cases younger than 15 years for whom the month of reporting was known, 1,235 (59.4%) were reported between August and October.

### Travel patterns

From 2009 to 2015, residents from the 13 participating countries spent ca 9.6 billion nights abroad (Table 2). The most visited regions were Europe (78.4% of all nights spent, including Turkey), America (8.5%) and Asia (7.4%). Travellers only spent 4.6% of their nights in Africa and 1.1% in Oceania. Travellers from France and Portugal spent a larger proportion (13.3% and 9.6%, respectively) of their nights in Africa compared with those from other countries. Travellers from Denmark, Finland and Norway spent 9.6% or more of their nights in Asia. 

**Table 2:** Number of nights spent on overseas trips, by reporting country and world region of destination, 13 EU/EEA countries^a^, 2009−2015 (n = 9,638 million)

Country	Nights spent on oversea trips					
	n (million)	Distribution by destination (%)				
		Africa	America	Asia	Europe	Oceania
Austria	469	3.8	6.6	6.8	81.9	0.9
Denmark	364	3.6	8.9	9.6	76.8	1.1
Estonia	52	3.1	18.5	7.1	71.3	No data
Finland	313	2.6	7.6	11.3	77.0	1.5
France	1,500	13.3	12.8	8.9	63.6	1.4
Germany	4,971	5.1	8.6	7.3	77.9	1.2
Greece	59	3.0	6.3	5.5	85.0	0.2
Hungary	187	2.1	9.8	4.6	82.4	1.1
Luxembourg	73	4.5	6.1	4.2	84.9	0.3
Malta	16	5.8	4.3	4.9	82.2	2.8
The Netherlands^b^	1,180	3.9	8.3	6.5	80.3	1.1
Norway^c^	369	4.2	8.2	9.6	77.0	1.0
Portugal	85	9.6	12.4	3.6	73.8	0.7
**Total**	**9,638**	**4.6**	**8.5**	**7.4**	**78.4**	**1.1**

^a^ Austria, Denmark, Estonia, Finland, France, Germany, Greece, Hungary, Luxembourg, Malta, the Netherlands, Norway, Portugal.

^b^ Number of nights only available for 2012−2015. The figure used is an estimate based on the average for available years.

^c^ Number of nights only available for 2009−2011. The figure used is an estimate based on the average for available years.

Within Europe, most nights were spent in the EU (84.5%). This proportion was below 80% for travellers from Estonia (71.5%) and Greece (58.4%). Overall, 63.8% of the nights spent in America were in North America. 

Nine of the 13 countries included had a risk between 0.20 and 0.46 cases per million nights. Travellers from France (1.67 cases per million nights), Greece (1.03), Estonia (0.87) and Portugal (0.73) had a higher risk (Table 3).

**Table 3:** Number of travel-associated cases of hepatitis A, number of nights spent and risk by reporting country, 13 EU/EEA countries, 2009−2015 (n = 5,233)

Country	Travel-associated cases			Nights (million)	Cases/million nights (95% CI)
	n	%	% <15 years		
Austria	96	1.8	36.5	469	0.21 (0.17–0.25)
Denmark	167	3.2	41.9	364	0.46 (0.39–0.53)
Estonia	45	0.9	4.4	52	0.87 (0.64–1.16)
Finland	78	1.5	38.5	313	0.25 (0.20–0.31)
France	2,510	48.0	43.1	1,500	1.67 (1.61–1.74)
Germany	1,619	30.9	37.1	4,971	0.33 (0.31–0.34)
Greece	61	1.2	34.4	59	1.03 (0.79–1.33)
Hungary	47	0.9	17.0	187	0.25 (0.19–0.33)
Luxembourg	15	0.3	33.3	73	0.20 (0.11–0.34)
Malta	5	0.1	20.0	16	0.32 (0.10–0.74)
The Netherlands ^a^	400	7.6	40.8	1,180	0.34 (0.31–0.37)
Norway ^b^	128	2.4	34.4	369	0.35 (0.29–0.41)
Portugal	62	1.2	33.9	85	0.73 (0.56–0.94)
**Total**	**5,233**	**100**	**39.8**	**9,638**	**0.54 (0.53–0.56)**

CI: confidence interval.

^a^ Number of nights only available for 2012−2015. The figure used is an estimate based on the average for available years.

^b^ Number of nights only available for 2009−2011. The figure used is an estimate based on the average for available years.

Of the 2,581 cases reported by 12 countries with information on probable country of infection (data from France excluded), 2,416 (93.6%) resided in the 10 countries for which travel data were available for all destinations (travel data for Estonia and Norway were not available for all destinations). Among these 2,416 cases, the overall risk for travel-associated hepatitis A was 0.31 cases per million nights spent overseas. The highest risks were observed with travel to Africa (2.11 cases per million nights) and Asia (1.25) (Table 4).

**Table 4:** Number of travel-associated cases of hepatitis A, number of nights spent and risk by travel destination, 10 EU/EEA countries^a^, 2009−2015 (n = 2,416)

Region	Travel-associated cases			Nights (million, estimate 2009−15^b^)		Cases/million nights (95% CI)
	n	%	% <15 years			
**Total (all regions)**	**2,416**	**100**	**39.8**	**7,794**	**100**	**0.31 (0.30–0.32)**
Africa	753	31.2	33.3	357	4.6	2.11 (1.96–2.27)
America						
North America	10	0.4	0.0	412	5.3	0.02 (0.01–0.04)
Central and South America^c^	106	4.4	14.2	228	2.9	0.46 (0.38–0.56)
Asia	705	29.2	50.4	564	7.2	1.25 (1.16–1.35)
Europe						
EU 28	427	17.7	13.6	5,150	66.1	0.08 (0.07–0.09)
Other European countries^d^	410	17.0	56.8	995	12.8	0.41 (0.37–0.45)
Oceania	5	0.2	0.0	88	1.1	0.06 (0.02–0.13)

CI: confidence interval.

^a^ Austria, Denmark, Finland, Germany, Greece, Hungary, Luxembourg, Malta, the Netherlands and Portugal.

^b^ The number of travel-associated cases was aggregated for the period 2009 to 2015, but travel nights were not available for all combinations of the different countries of residence with the different destination countries for all years. We therefore estimated the total number of nights from the mean number of nights for the available years multiplied by 7 (study period of 7 years)

^c^ Including Mexico.

^d^ Including Turkey.

Travel to Egypt, Turkey and Morocco accounted for 30.9% of all travel-associated cases reported with information on probable country of infection (Table 5). While most cases returning from Turkey and Morocco were reported in the period from August to October (64.2% and 62.6%, respectively), 47.7% of cases returning from Egypt were reported between November and February. Of note, of the 69 cases associated with travel to Italy, 24 were reported in 2013. The proportion of cases younger than 15 years was above 70% for cases reported with a travel to Afghanistan, Ethiopia, Iraq or Pakistan and below 10% for cases who travelled to France, Greece, Spain and Thailand. The proportion of cases younger than 15 years was noticeably low in travellers to Egypt (14.7%) and India (17.9%) compared with neighbouring countries such as Lebanon (45.0%) or Pakistan (73.6%). In the EU, this proportion was higher in travellers to Romania (29.4%), Bulgaria (22.6%) and Italy (20.3%) compared with other EU countries.

**Table 5:** Top destinations for travel-associated hepatitis A cases, 12 EU/EEA countries^a^, 2009−2015 (n = 2,581)

Rank	Destination country	Region	Travel-associated cases		
			n	%	% <15 years
1	Turkey	Other European countries	318	12.3	61.3
2	Egypt	Africa	279	10.8	14.7
3	Morocco	Africa	200	7.7	52.0
4	Pakistan	Asia	140	5.4	73.6
5	Afghanistan	Asia	130	5.0	80.0
6	India	Asia	106	4.1	17.9
7	Syria	Asia	92	3.6	52.2
8	Spain	EU 28	86	3.3	3.5
9	Italy	EU 28	69	2.7	20.3
10	Lebanon	Asia	60	2.3	45.0
11	Namibia	Africa	59	2.3	15.3
12	Romania	EU 28	51	2.0	29.4
13	Iraq	Asia	42	1.6	73.8
14	Bulgaria	EU 28	31	1.2	22.6
14	France	EU 28	31	1.2	6.5
16	Ethiopia	Africa	29	1.1	79.3
17	Peru	Central and South America	28	1.1	25.0
18	Croatia	Africa	26	1.0	26.9
19	Philippines	Asia	25	1.0	24.0
20	Greece	EU 28	24	0.9	8.3
20	Thailand	Asia	24	0.9	8.3
Other destinations			731	28.3	25.2
**Total**			**2,581**	**100**	**36.9**

^a^ Austria, Denmark, Estonia, Finland, Germany, Greece, Hungary, Luxembourg, Malta, the Netherlands, Norway and Portugal.

### Multivariable analysis

Male travellers were more likely than female travellers to have acquired their infection abroad (adjusted OR = 1.10; 95% CI: 1.02–1.18) (Table 1), and younger cases were more likely to be travel-associated than older cases. Compared with those aged 25–44 years, children younger than 5 years were 60% more likely to be reported with a probable infection abroad (adjusted OR = 1.59; 95% CI: 1.38–1.83).

## Discussion

### Principal findings

In the participating European countries, 27.8% of reported hepatitis A cases were travel-associated. Both this proportion and the number of travel-associated cases were stable over the study period. However, the risk for travel-associated hepatitis A per nights spent overseas decreased. This is consistent with decreases reported from the Netherlands [14] and Denmark in the 2000s [19]. The large number of cases reported in 2013 may have been related to a large multistate food-borne outbreak with more than 1,500 cases in the EU/EEA [20]. It is possible that other outbreaks also played a role in the fluctuations observed in these data. More than 60% of all travel-associated hepatitis A cases were associated with travel to Africa and Asia, although these destinations accounted for less than 15% of all nights spent overseas. These findings are hardly surprising because these two continents host most countries with high endemicity [4]. However, these estimates at continental level may mask important disparities. Some European countries are also at moderate to high risk for HAV infection. 

Age and seasonality appeared to be related to specific destinations. Pakistan was associated with a large proportion of cases younger than 15 years, most of whom were reported between August and October. These children were most probably accompanying their parents during the summer vacation. Conversely, Egypt, India and most European countries were associated with more cases among adults and a less pronounced seasonality. This may be suggestive of leisure travel without children or business travel which is year-round for some destinations. 

Interestingly, most countries of residency included in the analysis had comparable travel patterns and estimated risks, suggesting that some of these findings could be generalised to other European countries not included in this study. The higher risk observed in travellers from France and Portugal, compared with other countries, could be partly driven by a higher proportion of nights spent in Africa, a destination where travellers are at high risk of acquiring hepatitis A. Similarly, travellers from Estonia and Greece had a high proportion of nights spent in Eastern Europe and Turkey, which are destinations of higher risk compared with Western Europe [4].

### Strengths and weaknesses of the study

This study included more than 5,000 travel-associated hepatitis A cases reported by 13 countries. These countries account for more than 40% of the EU/EEA population. Our findings provide a good picture of the epidemiology of travel-associated hepatitis A in Europe in recent years. However, this study had some limitations. Firstly, a large proportion of mild non-specific or entirely asymptomatic hepatitis A cases, especially among children, were probably not captured by the surveillance systems. In addition, given the long incubation period of hepatitis A and the possibility of a case having visited several countries, countries may have misclassified some of their cases, and it is challenging to assess the effect of this type of error in our analysis. Secondly, information on the probable country of infection was not available for cases reported by France, which accounted for nearly half of travel-associated cases reported over the study period. Thirdly, we excluded countries with low coverage of their surveillance system (e.g. Italy) or low completeness of the travel status (e.g. the UK). Fourthly, nights spent by EU/EEA residents younger than 15 years were not available. Although children are unlikely to travel without their parents, we may have overestimated the risk in destinations with a large proportion of cases in children (e.g. Asia). Unfortunately, travel denominator data at country level were not available for most destination countries outside Europe. Lastly, there were limitations related to the travel data. These data were collected mostly via household survey [17] which is prone to recall bias. In addition, number of nights spent in destinations with few travellers (e.g. Oceania) can show high variations over time [17]. However, we think that overall, these data were reliable with little variation over the study period for most destinations considered in the analysis. 

Information on travel purpose and probable route of infection were not available. Some authors have recommended recording the travel purpose in order to inform public health strategies [21]. For example, people visiting friends and relatives are at a higher risk of acquiring travel-associated infection [22-24]. However, visiting friends and relatives may not be the sole purpose of the journey and the classical definition of visiting friends and relatives has been challenged [25]. Information on the probable route of infection may also be useful for informing prevention strategies as hepatitis A infection can also be sexually transmitted and men who have sex with men are a known risk group [26].

### Comparison with other studies

Our risk estimate (0.54 cases per million nights) is higher than data reported by Askling et al. who estimated a risk of travel-associated hepatitis A of 0.61 cases per 100,000 travel months (i.e. 0.20 cases per million nights) among Swedish travellers [13]. Our overall estimate was probably driven by France, a country whose travellers spent more nights in Africa and were shown to be less likely to be vaccinated compared with other European travellers [12]. As in our study, Askling at al. found a higher risk associated with travel to some parts of Africa and Asia. The proportion of travel-associated cases in our study (ca 30%) was lower than in the US (ca 40%) [10]. However, this overall proportion masks important disparities across countries and was heavily influenced by countries reporting many locally acquired cases such as Hungary. Alongside Romania and Slovakia, Hungary was one of the few EU/EEA countries with a notification rate above 10 cases per 100,000 population in 2014 [15]. In the late 1990s, routine hepatitis A vaccination of children was implemented in those states in the US with the highest incidence, leading to decreases in incidence among younger age groups [27]. It is possible that this strategy, implemented at local level, had a large impact on domestically acquired cases overall by decreasing HAV circulation in the US.

The high proportion of infection in children returning from countries less frequented by tourists (e.g. Afghanistan, Iraq or Pakistan) is very suggestive of travel to visit friends and relatives or recent immigration. This would confirm reports from Australia where travel-associated infections in children were more frequently associated with visiting friends and relatives than in adults [22]. The large number of refugees entering Europe in 2015 is unlikely to have had any major effect on our findings because reports from Germany and Greece suggested that hepatitis A cases in this population were only observed in late 2015 and 2016 [28,29].

### Possible explanations and implications for clinicians and policymakers

During the period from 2009 to 2015, the notification rate of hepatitis A cases in the EU/EEA was fairly stable at ca three cases per 100,000 population, with the highest notification rate observed in children aged 5–14 years. Our data suggest that the risk of travel-associated cases decreased slightly over the period. A molecular cluster analysis carried out in the Netherlands showed that travelling children were an important group for introduction and further transmission of HAV [30]. HAV vaccination, for children of families travelling to countries of high or intermediate endemicity would probably have a high impact on the number of travel-associated hepatitis A cases in Europe and consequently on secondary locally acquired cases. For example, HAV incidence in children of Moroccan or Turkish descent steadily declined after the introduction of targeted HAV vaccination programmes in the Netherlands [31]. More generally, hepatitis A vaccine is recommended for all non-immune travellers to countries of intermediate or high endemicity. Since a single dose of hepatitis A vaccine provides high protection even when given shortly before departure [32], any increase in opportunities for vaccination would be beneficial (e.g. vaccination clinics at airports). However, the recent vaccine shortages in some European countries may jeopardise efforts to promote vaccination [33]. Studies exploring other factors for not getting vaccinated (risk perception, cost etc.) would also help design effective public health measures [34].

## Conclusion

Travel remains a major risk factor for HAV infection in the EU/EEA, although the risk of infection may have slightly decreased over recent years. This still represents a considerable cause of morbidity which could be avoided since a safe, effective and affordable vaccine is available. Children younger than 15 years account for a large proportion of cases and should be prioritised for vaccination, particularly when travelling to visit relatives and friends in HAV-endemic countries.
